# The Effects of Honey Sulfonamides on Immunological and Hematological Parameters in Wistar Rats

**DOI:** 10.3390/medicina58111558

**Published:** 2022-10-30

**Authors:** Ionela Daniela Morariu, Liliana Avasilcai, Oana Cioanca, Branco-Adrian Morariu, Madalina Vieriu, Corneliu Tanase

**Affiliations:** 1 Department of Environmental and Food Chemistry, Faculty of Pharmacy, Grigore T Popa University of Medicine and Pharmacy Iasi, 16th University Street, 700115 Iasi, Romania; 2 Department of Pharmacognosy, Faculty of Pharmacy, Grigore T Popa University of Medicine and Pharmacy Iasi, 16th University Street, 700115 Iasi, Romania; 3 Faculty of Pharmacy, Grigore T Popa University of Medicine and Pharmacy Iasi, 16th University Street, 700115 Iasi, Romania; 4 Department of Analytical Chemistry, Faculty of Pharmacy, Grigore T Popa University of Medicine and Pharmacy Iasi, 16th University Street, 700115 Iasi, Romania; 5 Department of Pharmaceutical Botany, Faculty of Pharmacy, George Emil Palade University of Medicine, Pharmacy, Science and Technology of Targu Mures, 38th Gh. Marinescu Street, 540139 Targu Mures, Romania

**Keywords:** honey, immunologic, hematological, rats, sulfonamides

## Abstract

Sulfonamides are among the most used drugs in beekeeping due to their effectiveness, despite their long-term persistence in tissues. Bee honey containing such residues poses numerous risks to human health. The aim of the study was to evaluate the effects on immunological and hematological parameters of Wistar rats produced by sulfonamide residues in bee honey, through the evaluation of various blood parameters such as triiodothyronine and thyroxine levels, hematocrit, hemoglobin, red blood cell count and mean corpuscular hemoglobin concentration in a given volume of erythrocytes following administration of sulfonamide-containing honey. The hematological and immunological parameters showed significant variations in the group of rats that had been fed with honey spiked with sulfonamides compared to the control group. Changes in hematological indices were demonstrated in terms of a significant reduction in the number of erythrocytes, the amount of hemoglobin, and the value of hematocrit, thus confirming the induction of anemia in the tested group. Investigation of thyroid function through the analysis of triiodothyronine (T3) and thyroxine (T4) and their ratio showed a very significant decrease in plasma thyroxine levels in laboratory rats that were fed sulfonamide-spiked honey compared to the control group. The mean T3 concentration decreased from 0.70 ± 0.14 ng/dL to 0.34 ± 0.03 ng/dL, while the mean T4 concentration was reduced from 4.50 ± 0.30 μg/dL to 3.32 ± 0.21 μg/dL, thus demonstrating toxic effects on thyroid function. In sum, the presence of sulfonamides induced significant changes in the evaluated parameters indicating that the consumption of contaminated honey samples represents a high risk factor for thyroid dysfunction with potentially serious health impacts.

## 1. Introduction

Bee honey is one of oldest natural food items. It was considered a gift from God in ancient times, and it has been used as a therapeutic agent since the beginning of human civilization. Nowadays, honey is produced in a polluted environment and beekeeping practices include the use of various chemicals and drugs; thus, today’s bee honey contains a variety of these residues [1,2,3,4].

There are currently no maximum residue limits (MRLs) specified in the EU for sulfonamides (or other veterinary medicinal products) in honey; treatment of hives with these antimicrobials is prohibited, but these drugs can still be found in the final product produced by bees [5].

At present, sulfonamides are among the most used drugs in veterinary medicine due to their low price (compared to antibiotics), a broad antibacterial spectrum, and appreciable therapeutic efficacy against some infectious diseases. Sulfonamides are effective against bee diseases, but drug residues can persist in the tissues for a long period (sometimes up to a month) and the consumption of honey containing such residues poses risks to human health. The widespread use of such drugs in veterinary medicine represents a potential danger to human health as sulfonamides are considered to have average toxicity. Their presence in bee honey as residues may cause allergic reactions or antibiotic resistance phenomena in humans [6,7,8,9,10,11,12,13].

Various bioassays have shown that some sulfonamides cause tumors in different locations. In addition, sulfonamides can sometimes cause drug fever, serum sickness and systemic lupus erythematosus (type III hypersensitivity mediated by immunoglobulin G), and liver toxicity (including necrosis). Evidence of sulfonamide toxicity to the thyroid gland has also been reported [14]. Some sulfonamides inhibit lactoperoxidase and thyroid peroxidase, which are mediators in the synthesis of thyroid hormones, by competitive mechanisms, which can lead to hyperthyroidism.

The thyroid gland exerts essential regulatory influences on cell growth, on hormonal balance in general, on differentiation and metabolism, as well as on maintaining metabolic activity and affects the development of the entire skeletal system and other organs. Diseases that affect the normal functioning of the thyroid exhibit a wide range of symptoms, which can often be confused. Measurements of the thyroid hormones triiodothyronine (T3) and thyroxine (T4) by immunological methods are the most reliable tests for assessing the presence of thyroid dysfunction.

The objective of the study was to evaluate the influence of sulfonamide residues in bee honey on Wistar rats by quantifying immunological parameters, such as T3 and T4, and hematological parameters, such as hematocrit (Hct), hemoglobin (H), red blood cell count (RBC), and mean corpuscular hemoglobin concentration in a given volume of erythrocytes (MCHC) after administration of sulfonamide-containing honey.

## 2. Materials and Methods

### 2.1. Materials

For quantitative determination of the immunological parameters, the kit for the determination of T3 cod EH-500 and the kit for the determination of T4 cod EH-501 (produced by ClinPro International Co., LLC, Union City, CA, USA) were used. These contained 1 mL of calibrator solutions for the following concentrations 0, 0.75, 1.5, 3.0, 6.0, 10.0 ng/mL for T3 and 0, 2, 5, 10, 15, 25 μg/dL for T4, respectively.

Reagents used for the determination of eosinophils (hemoleukogram) were dedicated reagents for the automatic hematology analyzer Sysmex XT 1800i: Cellpack (diluent for hematological analyzers), the Stromatolyser FB (diluent for hematological analyzers), the Stromatolyser 4DL (diluent for hematological analyzers), the Stromatolyser 4DS (used for staining leukocytes from previously diluted and lysed samples), and Cellclean (strong alkaline detergent used to remove lysate residues, cell residues and proteins from the hydraulic system of the analyzer).

### 2.2. Experimental Protocol

For the study, Wistar female rats weighing 180–220 g were selected. The rats were divided into two groups of 10 rats each: a control group that were fed 2 mL of sulfonamide-free honey by gastric gavage and a sample group fed by gastric gavage for 5 days with honey spiked with 100 µg mixture per kilogram of body weight (µg/kgBW). The 100 µg mixture contained 20 µg of each of the five sulfonamides (i.e., sulfadiazine, sulfamethazine, sulfathiazole, sulfamethizole and sulfadimethoxine). All rats were individually housed under specific microclimate conditions and fed a standard plant-based diet with free water and food access

EU Regulation no. 37/2010 of the Commission has established a total maximum residue level of 100 µg/kg for all substances belonging to the sulfonamide group. This level applies to lean and fatty meat, liver, and kidney from all animals raised for consumption; it also applies to milk and honey [5].

All animal protocols were carried out in accordance with the instructions of the relevant guide regarding animal care and scientific use, in strict accordance with international ethical regulations [15,16]. The study was conducted in accordance with the 2010/63/EU directive and followed the recommendations of the National Institutes of Health (NIH) Guide for the Care and the Use of Laboratory Animals. Prior to the beginning of the study, the protocol received ethical approval from the University of Medicine and Pharmacy “Grigore T. Popa”, Iasi, Ethics Committee on 14 December 2011.

### 2.3. Sampling Protocol

Blood samples were collected in vacutainers containing anticoagulants (1% EDTA-Na_2_—1 part to 9 parts of whole blood) and were analyzed immediately to determine the hematological parameters (complete blood count). Blood samples collected in vacutainers without anticoagulant were immediately centrifuged and serum was sampled to determine the immunological parameters. Serum samples were kept frozen at −25 °C until testing.

### 2.4. Assay Protocol

The quantitative determinations of the immunological parameters (T3, T4) were performed on the Stat Fax 303 Plus device (Awareness Technology, Ramsey Minnesota, MI, USA), and of hematological parameters were performed on the automatic hematology analyzer, model Sysmex XT 1800i (Sysmex Europe Gmbh, Norderstedt, Germany).

Competitive-type ELISA was used for immunological determinations using a T3 determination kit, code EH-500 (ClinPro International Co., LLCClin Pro International, Union City, CA, USA) and a T4 determination kit, code EH-501 (ClinPro International Co., LLCClin Pro International, Union City, CA, USA).

### 2.5. Statistical Analysis

All experiments were performed in triplicate. All data are expressed as the mean ± standard deviation. The data obtained were statistically processed to eliminate biological variations and determination errors. For this purpose, statistical description of the samples using descriptors of interest (mean, median, standard error of the mean, standard deviation, variance, skewness coefficient, kurtosis coefficient) was performed.

## 3. Results

### 3.1. Immunological Results

A comparative analysis of the T4 results from the two groups showed a highly statistically significant *p* value that highlighted the presence of a marked decrease in T4 values in the sample group compared to the control group (Table 1). A comparative analysis showed that the T3 values of the sample group were significantly lower than those of the control group indicated by a statistically significant *p* value (Table 2).

### 3.2. Hematological Results

The results obtained for the hematological parameters following the administration of honey containing sulfonamide residues in Wistar rats are presented in Table 3. Analyzing the results, it was found that the values for Hct in the sample group were higher compared to the control group (Table 4).

The description and statistical analysis of the two groups for RBC showed a significant decrease in the values obtained in the sample group compared to the control group (Table 5).

For Hb, the data analysis highlighted changes of statistical significance. The values for the sample group were significantly lower than those for the control group (Table 6).

In terms of the MCHC values, it was found that there were statistically significant differences between the two groups. For the sample group, the values obtained for MCHC were lower than those of the control group (Table 7).

## 4. Discussion

Normal hematological values for Wistar rats are listed in Table 8. There are limited data in the literature on changes to hematological parameters resulting from toxicity produced by administration of honey containing sulfonamide residues.

The results of this study showed that the observed variations in hematological parameters in laboratory rats fed with sulfonamide-spiked honey showed decreased values for Hct, Hb, and MCHC. Among these, reduced values for erythrocyte count, the amount of hemoglobin and the value of hematocrit are the main parameters for the diagnosis of anemia. The scientific data indicate that 15% of the reported clinical cases of sulfonamide use have been associated with hematological changes (anemia, thrombocytopenia, neutropenia, etc.) in humans. These results concern adverse reactions to medication. No food-related sulfonamide contamination has so far been reported—contaminated batches have been disposed of prior to being consumed [18].

The results obtained for triiodothyronine (T3) values confirmed the hypothesis that sulfonamides interfere with the synthesis of thyroid hormones. For the group of animals given sulfonamide-spiked honey, the mean T3 concentration decreased from 0.70 ± 0.14 ng/dL to 0.34 ± 0.03 ng/dL. The intervention of sulfonamides in the metabolism of thyroid hormones resulted in changes in T3 concentrations which were uncorrelated with the quantity of sulfonamides administered with bee honey by gastric gavage in laboratory rats.

Plasma thyroxine (T4) levels decreased significantly in animals that were fed sulfonamide-spiked honey compared to the control group. These results are consistent with those published by Cribb et al. when evaluating the adverse effects of sulfonamides and sulfonamides/trimethoprim on rats [19].

In rats, administration of high doses of various sulfonamides has been associated with decreased T3 and T4 serum levels as well as increased TSH (thyroid stimulating hormone) levels [20]. These results are consistent with hypothyroidism reported in the literature in human studies as part of hypersensitivity reactions following treatment with one or more sulfonamides [21]. According to the same article, when administered to human subjects, the sulfonamides were converted by thyroid peroxidase into reactive metabolites that caused localized destruction of the thyroid gland. The thyroid lesions were found to be reversible after discontinuation of the drug.

The most important health concern associated with sulfonamide ingestion is thyroid adenoma. Chronic feeding (between 18 to 24 months) studies in rodents showed dose-dependent carcinogenic effects. As a result of these observations, the United States imposed an interdiction on the use of sulfonamides in cows above 20 months of age as such substances can transfer to milk as residues. A market analysis has indicated that more than 73% of tested milk samples contained sulfonamides [18]. From a biological point of view, the toxicity of substances is not the same for all species of living organisms. This is a result of the different rates and ways of eliminating the toxic metabolite, as well as of the different sensitivities of the species to that metabolite. Observed differences between species are primarily due to metabolic factors, which, in turn, depend on the enzymes that control the biotransformation of the toxic substance. Such differences reflect differences between different species in terms of the kinetic parameters of transport, distribution, storage, redistribution and blood/tissue distribution, as well as differences in the methods and rates of bio-inactivation or elimination of the toxic substance.

## 5. Conclusions

Changes in hematological indices were demonstrated in terms of a significant reduction in the number of erythrocytes, the amount of hemoglobin, and the value of hematocrit. Investigation of thyroid function through analysis of T3 and T4, and their ratio, showed a very significant decrease in plasma thyroxine levels in laboratory rats that were fed sulfonamide-spiked honey compared to a control group.

Reducing the content of sulfonamide residues in honey and other bee products can be achieved by abiding by the recommended time period between the administration of chemotherapy and harvesting of the bee product, as well as by reducing the frequency of application of veterinary treatments.

Our research suggests the potential for the quantification and individual identification of other antimicrobial substances, as well as other pollutants (pesticides, toxic metals, biostimulators, mycotoxins), used in the treatment of diseases in bees and which are found as residues in honey. It also highlights the importance of the evaluation of changes in biochemical and hematological parameters and oxidative stress when investigating the effects of metabolite residues resulting from the administration of bee honey and other bee products (e.g., pollen, royal jelly, propolis) as well as other food products.

## Figures and Tables

**Table 1:** Data processing of thyroxine values (T4 as µg/dL).

Parameter	Control Group	Sample Group
Mean ± SD	4.50 ± 0.30	3.32 ± 0.21
Mean ± SE	4.50 ±0.13	3.32 ± 0.09
Median	4.33	3.34
Standard deviation	0.30	0.21
Variance	0.04	0.09
Skewness coefficient	0.81	0.55
Kurtosis coefficient	−1.54	−0.41
Amplitude	0.70	0.52
Minimum value	4.22	3.10
Maximum value	4.92	3.62
Number of replicates (n)	5	5
Confidence level (95%)	0.37	0.26
Confidence interval (95%)	4.12–4.87	3.06–3.58
Kolmogorov–Smirnov Test	*p* 0.98 0.63	
Student’s *t*-test	t (t stat) t (t critic)	*p* (T ≤ t) *p* (F-test)
Control vs. Sample Group	−7.21 1.86	<0.001 * 0.49 4.58·10^−5^

(*) *p* < 0.05 significant, *p* ≤ 0.01 distinctly significant, *p* ≤ 0.001 very significant.

**Table 2:** Data processing of triiodothyronine values (T3 as ng/dL).

Parameter	Control Group	Sample Group
Mean ± SD	0.70 ± 0.14	0.34 ± 0.03
Mean ± SE	0.70 ± 0.06	0.34 ±0.02
Median	0.69	0.35
Standard deviation	0.14	0.03
Variance	0.001	0.02
Skewness coefficient	−0.15	−0.84
Kurtosis coefficient	−1.70	0.70
Amplitude	0.34	0.09
Minimum value	0.52	0.29
Maximum value	0.86	0.38
Number of replicates (n)	5	5
Confidence level (95%)	0.17	0.04
Confidence interval (95%)	0.53–0.88	0.30–0.38
Kolmogorov–Smirnov Test	*p* 0.98 0.97	
Student’s *t*-test	t (t stat) t (t critic)	*p* (T ≤ t) *p* (F-test)
Control vs. Sample Group	−5.57 2.13	0.003 * 0.018

(*) *p* < 0.05 significant, *p* ≤ 0.01 distinctly significant, *p* ≤ 0.001 very significant.

**Table 3:** Hematological parameters after administration of honey with sulfonamide residues to rats.

Parameter	Mean ± SD	
	Control Group	Sample Group
Hct (%)	47.80 ± 3.69	52.70 ± 6.64
RBC (million/mm^3^)	9.72 ± 1.14	5.14 ± 0.38
Hb (g/dL)	16.10 ± 0.92	14.50 ± 1.16
MCHC (g/dL)	38.60 ± 2.79	34.50 ± 2.20

Hct—hematocrit, Hb—hemoglobin, RBC—red blood cell count, MCHC—mean corpuscular hemoglobin concentration in a given volume of erythrocytes.

**Table 4:** The data processing of hematocrit (Hct) values.

Parameter	Control Group	Sample Group
Mean ± SE	47.80 ± 1.65	52.70 ± 2.97
Median	47.36	50.16
Standard deviation	3.69	6.64
Variance	13.61	44.03
Skewness coefficient	−0.30	0.71
Kurtosis coefficient	1.12	−0.98
Amplitude	10.15	16.40
Minimum value	42.47	45.76
Maximum value	52.62	62.16
Number of replicates (n)	5	5
Confidence level (95%)	4.58	8.24
Confidence interval (95%)	43.22–52.38	44.46–60.94
Kolmogorov–Smirnov Test	*p* 0.94 0.86	
Student’s *t*-test	t (t stat) t (t critic)	*p* (T ≤ t) *p* (F-test)
Control vs. Sample Group	−1.44 1.86	0.09 * 0.28

(*) *p* <0.05 significant, *p* ≤ 0.01 distinctly significant, *p* ≤ 0.001 very significant.

**Table 5:** Data processing of red blood cell count values (RBC as million/mm^3^).

Parameter	Control Group	Sample Group
Mean ± SE	9.72 ± 0.51	5.14 ± 0.17
Median	9.86	4.93
Standard deviation	1.14	0.38
Variance	1.31	0.15
Skewness coefficient	−0.13	1.02
Kurtosis coefficient	0.53	−0.76
Amplitude	3.10	0.88
Minimum value	8.14	4.83
Maximum value	11.24	5.71
Number of replicates (n)	5	5
Confidence level (95%)	1.42	0.48
Confidence interval (95%)	8.30–11.14	4.66–5.62
Kolmogorov–Smirnov Test	*p* 0.99 0.64	
Student’s *t*-test	t (t stat) t (t critic)	*p* (T ≤ t) *p* (F-test)
Control vs. Sample Group	8.49 1.86	<0.001 * 1.42·10^−5^ 0.057

(*) *p* <0.05 significant, *p* ≤ 0.01 distinctly significant, *p* ≤ 0.001 very significant.

**Table 6:** Data processing of hemoglobin values (Hb as g/dL).

Parameter	Control Group	Sample Group
Mean ± SE	16.10 ± 0.41	14.50 ± 0.52
Median	16.47	14.86
Standard deviation	0.92	1.16
Variance	0.85	1.34
Skewness coefficient	−1.34	−0.41
Kurtosis coefficient	1.19	−0.14
Amplitude	2.19	3.05
Minimum value	14.62	12.87
Maximum value	16.81	15.92
Number of replicates (n)	5	5
Confidence level (95%)	1.15	1.44
Confidence interval (95%)	14.96–17.25	13.06–15.94
Kolmogorov–Smirnov Test	*p* 0.85 0.94	
Student’s *t*-test	t (t stat) t (t critic)	*p* (T ≤ t) *p* (F-test)
Control vs. Sample Group	2.42 1.86	0.02 * 0.67

(*) *p* <0.05 significant, *p* ≤ 0.01 distinctly significant, *p* ≤ 0.001 very significant.

**Table 7:** Mean corpuscular hemoglobin concentration in a given volume of erythrocytes (MCHC as g/dL).

Parameter	Control Group	Sample Group
Mean ± SE	38.60 ± 1.25	34.50 ± 0.98
Median	37.07	34.58
Standard deviation	2.79	2.20
Variance	7.77	4.82
Skewness coefficient	0.65	−0.71
Kurtosis coefficient	−2.72	−0.03
Amplitude	5.94	5.56
Minimum value	36.24	31.25
Maximum value	42.18	36.81
Number of replicates (n)	5	5
Confidence level (95%)	3.46	2.73
Confidence interval (95%)	35.14–42.06	31.78–37.23
Kolmogorov–Smirnov Test	*p* 0.64 0.99	
Student’s *t*-test	t (t stat) t (t critic)	*p* (T ≤ t) *p* (F-test)
Control vs. Sample Group	2.58 1.86	0.02 * 0.66

(*) *p* <0.05 significant, *p* ≤ 0.01 distinctly significant, *p* ≤ 0.001 very significant.

**Table 8:** Normal values of hematological parameters for Wistar rats [17].

Hematological Parameter	Unit of Measurement	Range of Variation
RBC	millions/mm^3^	5.5–10
Hct	%	53–60
Hb	g/dL	14–16
MCHC	g/dL	31–33
MCHC	pg/cell	17–20

Hct—hematocrit, Hb—hemoglobin, RBC—red blood cell count, MCHC—mean corpuscular hemoglobin concentration in a given volume of erythrocytes.
